# Urethral duplication associated with epispadias: Case report

**DOI:** 10.1016/j.ijscr.2024.110362

**Published:** 2024-09-27

**Authors:** Tafese Gudissa Merga, Hana Abebe Gebreselassie, Mohamed Ahmed, Mensur Mohammed, Hiwote Girma

**Affiliations:** a Pediatric Surgery Unit, Department of Surgery, St. Paul's Hospital Millennium Medical College, Addis Ababa, Ethiopia; b Urology Surgery Unit, Department of Surgery, St. Paul's Hospital Millennium Medical College, Addis Ababa, Ethiopia

**Keywords:** Urethral duplication, Epispadias, Urinary incontinence

## Abstract

**Introduction and importance:**

Urethral duplication is a rare congenital urinary tract anomaly. Its association with male epispadias is extremely rare. Due to various anatomical types, patients with urethral duplication can have a wide range of clinical presentations. Managing epispadias associated with urethral duplication involves different techniques and one should identify the functional urethra before proceeding with the surgery. It is associated with better functional outcomes than bladder epispadias exstrophy.

**Case presentation:**

We report a case of a 4-year-old male child presented with a complaint of urinary incontinence. After evaluation, he was diagnosed with Urethral duplication associated with penopubic epispadias. Epispadias repair with urethral advancement was done and the child had a smooth postoperative course.

**Clinical discussion:**

Both urethral duplication and epispadias are rare congenital urinary tract anomalies. Urethral duplication associated with epispadias is extremely rare. Patient clinical presentation varies from incontinence to incidental findings. The diagnosis of urethral duplication in epispadias patients is usually missed preoperatively and even intraoperatively. The type of procedure going to be done depends on the type of urethral duplication. Management of these patients is associated with good functional outcomes.

**Conclusion:**

Even though urethral duplication associated with epispadias is a rare congenital malformation, it should be carefully searched in male epispadias.

## Highlights

• Urethral duplication, a rare congenital anomaly, can present with varied symptoms; association with epispadias is extremely rare.
• Surgery involved epispadias repair with urethral advancement, resulting in a smooth postoperative course for the child.
• Identification of the functional urethra is crucial in managing epispadias with urethral duplication for better outcomes.
• Despite its rarity, urethral duplication with epispadias should be considered in male epispadias cases for proper management.

## 1 Introduction

Urethral duplication associated with epispadias is an extremely rare congenital urinary tract malformation [1,2]. Since it involves various anatomic ranges, patients with urethral duplication have different clinical presentations ranging from urinary incontinence to asymptomatic patients [1,3,4]. Managing urethral duplication associated with epispadias involves excision of the accessory urethra with preservation of functional urethra or epispadias repair with urethral advancement [[5], [6], [7]].

We are presenting a 4-year-old male child presented with urinary incontinence and diagnosed with urethral duplication associated with epispadias. This work has been reported following the SCARE criteria.

## 2 Case presentation

**Image 1:**
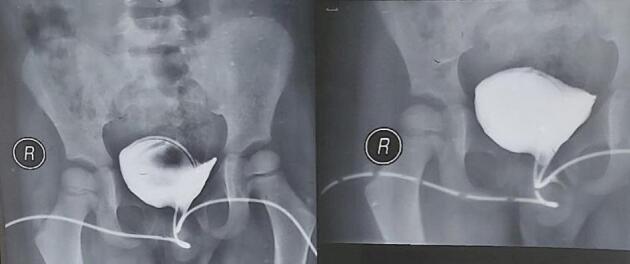
Cystourethrography which is taken preoperatively showing the two urethras are catheterized and independently entering a single urinary bladder.

**Image 2:**
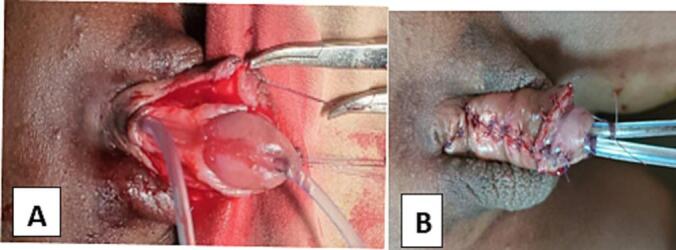
Intraoperative images showing two urethral openings catheterized with feeding tube 8F (penopubic Epispadias urethra and glanular located urethra). A. Before repair, just after the procedure is started. B. After Epispadias repair with urethral advancement.

A 4-year-old male child presented with a complaint of urinary incontinence. He has dorsally located urethral meatus and abnormal penile curvature since birth. The prenatal history was unremarkable. He was toilet trained at the age of three years but he had no dry time and continued to have continuous urine leaks. Otherwise, he has no other urinary complaint or history of urinary tract infections. On examination the scrotum is well-developed, and tests are palpable in the scrotum bilaterally. There were Penopubic epispadias (dorsal urethral meatus) with moderate dorsal chordae. Well-developed glans with another normally located urethral meatus (ventral urethral meatus) on the glans. Cystourography was done and showed two urethrae independently entering a single bladder (Image 1). Both urethrae were catheterized with an 8F feeding tube (Image 2A). The epispadias urethral plate was mobilized up to the coronal sulcus area. Then, it tubularized over the tube. Glans wing developed dorsally. The glanular part of the septum between the normally located urethra and the epispadias urethral plate is divided and the two urethra are made to communicate with each other at the glans. Urethroplasty was done over the tubes. Corporoplasty was done and dorsal curvature was corrected. Skin mobilized dorsally and approximated (Image 2B). Trans urethral tubes were removed after ten days and the child is having a smooth postoperative course at 6 months of follow-up.

## 3 Discussion

Urethral duplication is a rare congenital urinary tract malformation. It can occur in the sagittal plane (where the two urethrae are in the same sagittal plane, one above the other) or coronal plane (the urethras lie collaterally, side by side). The sagittal plane is the commonest one [1,4,8] Urethral duplication involves a wide range of anatomic variants and there are different classification systems, with Effman classification being commonly used [4,9]. The clinical presentation varies depending on the type of duplication and associated anomalies [4,8,10,11]. The patient can be incontinent, having just urinary dribbling, no urinary complaint but having two urethral openings with or without a double urinary stream [4,[11], [12], [13]], difficult during sexual intercourse, genital area discharge [3,[13], [14], [15]]. It can also be incidentally detected during urinary tract workup or surgery [12,14,16] or even may be missed at the time of surgery especially those associated with bladder exstrophy epispadias complex [17].

Isolated epispadias, which is relatively more common than epispadias associated with urethral duplication, is a rare exstrophy epispadias complex anomaly [14,17]. Epispadias associated with Urethral duplication are extremely rare anomalies [11,12,16,18]. The diagnosis of urethral duplication in these patients is usually missed preoperatively and the diagnosis will made intraoperatively [1,7,19]. In these patients, the ventral urethra is almost always located in the sphincter complex and is the functional one [11,12,20].

The management of urethral duplication is indicated in symptomatic patients, associated anomalies, or cosmetic deformities [4,9]. Managing epispadias associated with urethral duplication involves excision of the dorsal urethra with preservation of the ventral one [5,21]. One can do also epispadias repair with urethral advancement to connect the two urethral openings in case of type IIA urethral duplication associated with epispadias [6]. In the case of Y-type urethral duplication, multiple staged surgery may be needed to achieve the final complete result [3,8,22] One should identify the functional urethra before proceeding with the surgery [4,7,8,18]. The management outcome of epispadias associated with urethral duplication is associated with good functional outcomes [12,18], having better outcomes than bladder epispadias exstrophy [21,23,24].

## 4 Conclusion

Even though it is rare to find urethral duplication associated with epispadias, it should be carefully searched in male epispadias. One should identify the functional and accessory urethra before embarking on surgery.

## Ethical approval

Ethical approval is held to be unnecessary by St. Paul's Hospital Millennium Medical College Institutional Review Board as this is a single rare case encountered during clinical practice.

## Funding

No specific grant from funding organizations in the public, private, or nonprofit sectors was given to this manuscript.

## Author contribution

All authors contributed to different aspects. Hiwote Girma and Hana Abebe G/Sillassie operated on the patient and also following the patient. Mensur Mohammed and Mohammed Ahmed wrote the case presentation and wrote the draft of the case report. Tafese Gudissa wrote the final case report and is also following the patient. All authors read and approved the final manuscript.

## Guarantor

Tafese Gudissa Merga.

## Registration of research studies

Not applicable.

## Consent

A written informed consent has been obtained from the patient's guardian (the father) to have the case details and any accompanying images published. A copy of the written consent is available for review by the Editor-in-Chief of this journal on request.

## Declaration of competing interest

No authors have disclosed any conflicts of interest.
